# A Masked Aversive Odor Cannot Be Discriminated From the Masking Odor but Can Be Identified Through Odor Quality Ratings and Neural Activation Patterns

**DOI:** 10.3389/fnins.2019.01219

**Published:** 2019-11-14

**Authors:** Rea Rodriguez-Raecke, Helene M. Loos, Rik Sijben, Marco Singer, Jonathan Beauchamp, Andrea Buettner, Jessica Freiherr

**Affiliations:** ^1^Diagnostic and Interventional Neuroradiology, University Hospital, RWTH Aachen University, Aachen, Germany; ^2^Sensory Analytics, Fraunhofer Institute for Process Engineering and Packaging IVV, Freising, Germany; ^3^Chair of Aroma and Smell Research, Department of Chemistry and Pharmacy, Friedrich-Alexander-Universität Erlangen-Nürnberg, Erlangen, Germany; ^4^Scent & Care, Symrise AG, Holzminden, Germany; ^5^Department of Psychiatry and Psychotherapy, Friedrich-Alexander-Universität Erlangen-Nürnberg, Erlangen, Germany

**Keywords:** odor mixture, odor masking, fMRI, piriform cortex, ventral striatum

## Abstract

Odor masking is a very prominent problem in our daily routines, mainly concerning unpleasant sweat or toilet odors. In the current study we explored the effectiveness of odor masking both on a behavioral and neuronal level. By definition, participants cannot differentiate a fully masked unpleasant odor from the pleasant pure odor used as a masking agent on a behavioral level. We hypothesized, however, that one can still discriminate between a fully masked odor mixture and the pure masking odor on a neuronal level and that, using a reinforcing feedback paradigm, participants could be trained to perceive this difference. A pleasant, lemon-like odor (citral) and a mixture of citral and minor amounts of an unpleasant, goat-like odor (caproic acid) were presented to participants repeatedly using a computer-controlled olfactometer and participants had to decide whether two presented stimuli were the same or different. Accuracy of this task was incentivized with a possible monetary reward. Functional imaging was used throughout the task to investigate central processing of the two stimuli. The participants rated both stimuli as isopleasant and isointense, indicating that the unpleasant odor was fully masked by the pleasant odor. The isolated caproic acid component of the mixture was rated less pleasant than the pleasant odor in a prior experimental session. Although the masked and pure stimuli were not discriminated in the forced-choice task, quality ratings on a dimensional scale differed. Further, we observed an increased activation of the insula and ventral striatum/putamen for the pure in contrast to the fully masked odor, hence revealing a difference in neuronal processing. Our hypothesis that perceptual discrimination and neuronal processing can be enhanced using a reinforcing feedback paradigm is not supported by our data.

## Introduction

The process of human chemosensation can be tuned in manifold ways. At the peripheral level, olfactory receptors are specific to distinct odorants, but their affinity for odors can differ (Malnic et al., 1999). At a central level, the perceived odor is processed in the mitral cells of the olfactory bulb and can be inhibited or excited by neighboring cells and might be modulated in the piriform, orbitofrontal and insular cortices as well (Mori et al., 1999). The multitude of physiological processes involved in the process of olfaction and its modulating factors may explain why humans are able to improve olfactory abilities with practice (Li et al., 2006; Porter et al., 2007; McGann, 2017; Reichert and Schopf, 2017; Sorokowska et al., 2017). The olfactory neuronal interplay enables even subjects with a partial loss of their sense of smell to learn to detect odors after training using repetitive exposure (Wysocki et al., 1989; Boulkroune et al., 2007). Besides odor detection, odor discrimination may be improved by learning. Li et al. (2008) demonstrated that participants learned to discriminate indistinguishable odors by aversive conditioning involving the use of electric shocks. The authors used two pure odorants – two enantiomers that only differed in their chiral property. These enantiomers are very difficult to distinguish, and one of them was used as conditioned stimulus (CS+), which was presented with a higher probability concurrently with an electric shock. Distinct changes in spatially distributed patterns of neural activity in the piriform cortex during this task were observed.

Whereas distinguishing two similarly smelling pure odorants is one possible task for odor discrimination, another challenge is to distinguish between similarly smelling odor mixtures, or between a pure odorant and an odor mixture dominated by the same odorant. This latter task might be more relevant in real-life scenarios, since we are often surrounded by odor mixtures, and in public locations, or at home, pleasant odors may be used to mask aversive odors (Schifferstein et al., 2011). Usually, a large amount of the masking odor is needed to mask an unpleasant, e.g., body odor. Still, the masked aversive odor may be noticed due to a different percept or odor quality of the mixture, even though it may not be perceived directly. Compared to single odorants, odor mixtures represent a different concept on a perceptual level and are processed differently (Boyle et al., 2009); a masked odor falls in the latter category. Generally, odor mixtures can be processed in an elemental or configural way (Sinding et al., 2013). Most mixtures including up to three odors are processed elementally and more complex mixtures are processed configurally (Deisig et al., 2006, 2010). Accordingly, humans can differentiate not more than three to four odors in mixtures (Laing and Francis, 1989; Jinks and Laing, 2001). Quality, quantity, and temporal processing of odors (Jinks and Laing, 1999) are compelling factors for odor perception in mixtures, as are their characteristics as good or poor blenders (Livermore and Laing, 1998). In odor masking, a pleasant odor is used to mask an unpleasant odor, and it is expected that the unpleasant odor is completely covered by the masking odor. Nonetheless, neuronal processing is expected to differ between the pure masking odor and the odor mixture (Grabenhorst et al., 2007, 2011).

We aimed to explore whether reinforcement by reward improves odor discrimination of a masked stimulus, as positive feedback and reinforcement with a possible monetary reward have previously been shown to alter human behavior (Kirsch et al., 2003). We are addressing the question of whether a low concentration of a distinct but masked odor can be detected within an odor mixture. We hypothesized that the pure and the masked odor stimuli are not discriminated on a perceptual level, but that neuronal processing differs between the stimuli. Further, we hypothesized that perceptual discrimination can be enhanced using a positive reinforcing feedback paradigm.

## Materials and Methods

### Participants

Of the initially recruited 30 subjects, one subject was excluded due to technical problems, leaving a sample size of 29 healthy volunteers with a mean age of 26.86 years (SD = 4.37 years, range = 20–36 years, 11 females). All participants were normosmic, as confirmed by assessment during a screening phase prior to the experiment using the three-alternative forced-choice “Sniffin’ sticks” discrimination test (Kobal et al., 1996) with 16 odor triplets (mean = 13.17, SD = 1.8, range 9–16, missing data in five subjects) and the four-alternative, forced-choice MONEX-40 identification test (Freiherr et al., 2012) with 40 common odors (mean = 32.62, SD = 2.49, range 27–37). The study protocol was approved by the ethical review board of the Medical Faculty of RWTH Aachen University and all subjects gave written informed consent prior to inclusion in the study. All procedures and experiments were explained and conducted in accordance with the Declaration of Helsinki. Participants were free to withdraw from the experiment at any time. Exclusion criteria were neurologic diseases, psychiatric disorders, diseases of the nose or lungs, and smoking. Electronic versions of Beck’s Depression Inventory (BDI) (Beck et al., 1961; Ambrosini et al., 1991) and the Brief Symptom Inventory (BSI) (Derogatis and Melisaratos, 1983), as well as the Montreal Cognitive Assessment (MoCA) (Nasreddine et al., 2005), were completed by the subjects prior to participation. All participants fulfilled the inclusion criteria (BDI ≤ 13, BSI cut-off was based on the age and gender-based SCL-90-R norm values with GSI ≤ 60, MoCA ≥ 26).

### Experimental Design

#### Odor Stimuli

**FIGURE 1:**
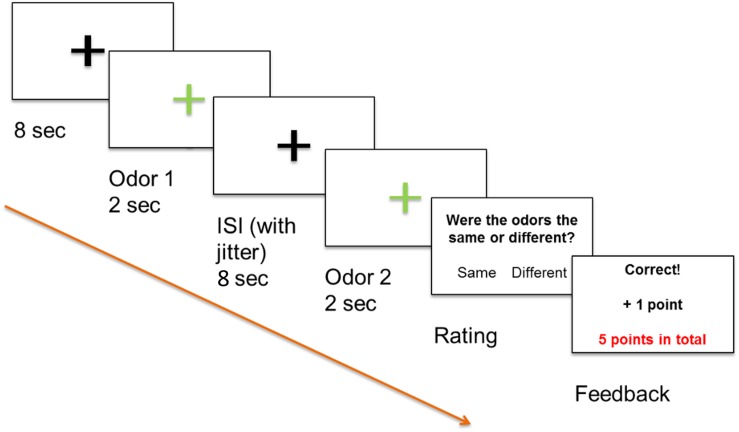
Positive feedback paradigm, odor 1 and odor 2 were presented sequentially and participants decided if the odors differed or not by pressing a button. All participants were informed that a high score increased the likelihood that they would receive a monetary reward.

Odor stimuli were applied via Teflon tubing gated to the nose of the participant inside the MRI scanner using a computer-controlled olfactometer (Lundstrom et al., 2010). At the end of the tubing, custom-made nose pieces were attached to the tubing. The participants were exposed to the odors once in advance of the experiment and carefully trained how to rate the quality of the odors. We chose the unpleasant odor component caproic acid to mimic an unpleasant sweat odor and chose the pleasant odor citral to mimic a cleaning substance. Odor stimuli consisted of 80% v/v citral (product-no.: 197010, Symrise AG, Holzminden, Germany) in propylene glycol (PG) (stimulus: citral) and a mixture of 80% v/v citral and 4% v/v caproic acid (product-no.: 182737, Symrise AG, Holzminden, Germany) in PG (stimulus: mix 1) and were applied for 2 s with an inter stimulus interval of at least 8 s, depending on the duration of the rating procedure. A mixture of 5% v/v citral and 4% v/v caproic acid in PG was used as a distractor stimulus (stimulus: mix 2). Pure PG was used as an odorless stimulus and was applied between odor trials, also covering the feedback condition (experimental design is depicted in Figure 1). Consistency of odor stimulation was assessed by on-line chemical analysis using proton transfer reaction-mass spectrometry (PTR-MS). This technique has been described in detail elsewhere and has been previously used for validating odor stimuli for olfactory perception analyses (Beauchamp et al., 2010; Denzer et al., 2014; Denzer-Lippmann et al., 2017). The gas-phase volume mixing ratios (concentrations) of citral generated by the olfactometer from these solutions were 2.03 ± 0.19, 1.91 ± 0.20, and 0.14 ± 0.02 ppmv for citral, mix 1, and mix 2, respectively, as quantified by PTR-MS (see Supplementary Materials).

#### Procedure

Functional magnetic resonance imaging (fMRI) was used in combination with Presentation software (Neurobehavioral Systems, Albany, CA, United States) and an event-related stimulation design. The experiment was run via the software and displayed on a computer screen (40″ 4K UHD InroomViewingDevice, NordicNeuroLab AS, Norway) that was visible to the subjects lying in the MRI scanner via a mirror. Our odor discrimination paradigm involved presentation of two odors in succession as an odor pair to both nostrils with a minimal inter-stimulus-interval (ISI) of 8 s to prevent habituation (Hummel et al., 1996). Odor stimuli were applied for 2 s via a computer-controlled olfactometer (Lundstrom et al., 2010) with a constant air flow of 3.0 l/min. Glass jars containing the three odor solutions (citral, mix 1, and mix 2), as well as PG as odorless stimulus, were installed in the olfactometer. Stimuli of pairs of the same odor or differing odors were applied in a pseudorandomized order. The task of the participants was to use an MRI-compatible keyboard to decide whether the odor pairs perceptually differed or not. A decision was required immediately after presentation of the second odor, with participants subsequently receiving an immediate feedback on the computer screen. Participants were informed that they would gain points for correct answers, lose points for incorrect answers, and that a high score increased the likelihood that they would receive a monetary reward. The paradigm is depicted in Figure 1. Twenty-five pairs with “same” stimuli, 25 pairs with “different” stimuli, and five pairs containing the distractor with “different” stimuli were applied, resulting in a total of 110 odor stimuli.

### Data Acquisition and Statistical Analysis

#### Behavioral Data

All calculations were performed using SPSS 22.0 (SPSS Inc., Chicago, IL, United States). Intensity and pleasantness of the odor stimuli were assessed during the functional imaging session using two visual analog scales (VAS, 0–100) ranging from “not perceivable” to “very strongly perceivable” and from “very unpleasant” to “very pleasant.” Odor quality ratings were acquired using a scale ranging from 100% goat (caproic acid, coded with 0) to 100% lemon (citral, coded with 100). Participants were instructed to rate a stimulus being in the middle of this scale (coded with 50) when they did not perceive any odor. A 3 × 5 ANOVA for the factors scale (pleasantness, intensity, quality) and odor (Baseline, caproic acid, citral, mix 1, mix 2) were conducted. Bonferroni-corrected *t*-tests comparing the pure citral stimulus with the fully masked stimulus (mix 1) as well as comparing citral and caproic acid were conducted for the scales of intensity, pleasantness and, quality. In accordance with the signal detection theory the parameters *d*′ and *c* (Velden, 1982) were calculated for each subject as a measure of their sensitivity to the task. Correlations of odor identification (MONEX-40), odor discrimination (Sniffin’ Sticks), *d*′ and achieved points were calculated. Accuracy against chance level (0.5) was also tested using a one sample *t*-test. A possible effect of improvement in the task was evaluated by comparing the first half of the experiment with the second half of the experiment. Again with “accuracy” as outcome measure, a paired *t*-test with part (first half/part 1, second half/part 2) was conducted. Further, we conducted a *post hoc* sensitivity analysis with G^∗^Power and found that our study was sufficiently sensitive [*t*-test, difference between two dependent means, α error probability = 0.05, total sample size = 29, assuming 1 – β error probability = 0.8 (Cohen, 1988)] to detect a medium to small effect size *dz* = 0.47 and a critical *t* = 1.70. We also conducted a *post hoc* power analysis with G^∗^Power, assuming a medium effect size (Hermann et al., 2000; Cook et al., 2018), *t*-test, *dz* = 0.5, means, within subject, total sample size = 29, α error probability = 0.05, critical *t* = 1.70) and found that the experiment is sufficiently powered with an achieved power (1 – β err probability) = 0.84. All data are reported as mean ± standard error of the mean (SEM). Significant results were considered as *p* < 0.05.

### Imaging Data

Functional MRI data were acquired using a 3 Tesla MRI scanner (Siemens Magnetom Prisma) with a 20-channel head coil. A T2^∗^-sensitive EPI (Echoplanar imaging) sequence was used, with 36 axial slices and a 20% distance factor, a matrix size of 80 × 80 mm, a voxel size of 2.5 × 2.5 × 2.5 mm, a FoV of 200 × 200 mm, TR of 2 s, TE of 30 ms, and a 77° flip angle. For structural image acquisition, a high-resolution T1 MPRAGE (Magnetization Prepared Rapid Gradient Echo) sequence was used, with 176 slices and a matrix size of 256 × 256 mm, a voxel size of 1.0 × 1.0 × 1.0 mm^3^, a FoV of 256 × 256 mm, TR of 2 s, TE of 2.28 ms, and a flip angle of 8°. Overall acquisition time was approximately 30 min. In total, 663 to 749 volumes were acquired per subject, depending on duration of the rating procedure.

Functional images were preprocessed and analyzed statistically with SPM12^1^ based on MATLAB (MathWorks, Natick, MA, United States). For data analysis, the approach of the general linear model (GLM) was used, modeling the correct and incorrect odor (citral, mix 1, and mix 2) and feedback events in a first-level design matrix for each subject. An event-related design with adjusted event durations was used, as durations for the feedback event varied, and in a separate model linear parametric modulation was used to reveal if activation due to the odors increased over the time course of the experiment. Additionally, realignment parameters were added as regressors of no interest. Difference images contrasting citral and mix 1 were also determined, as were those contrasting the first versus the second presented odor using either a one sample *t*-test or a paired comparison. Results were considered relevant with a *p* < 0.05 family-wise-error (FWE) correction for whole-brain comparison on the cluster level. Activated brain areas were defined using the SPM Anatomy toolbox (Eickhoff et al., 2005) and the Mai-Atlas (Mai et al., 2008) and were visualized using MRIcron software (Rorden et al., 2007).

## Results

### Behavioral Data

**FIGURE 2:**
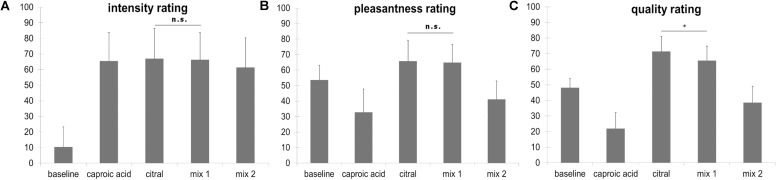
**(A)** Intensity ratings ranging from “not perceivable” (coded with 0) to “very strongly perceivable” (coded with 100), mix 1: fully masked stimulus, mix 2: incompletely masked stimulus. **(B)** Pleasantness ratings ranging from “very unpleasant” (coded with 0) to “very pleasant” (coded with 100), mix 1: fully masked stimulus, mix 2: incompletely masked stimulus. **(C)** Quality ratings ranging from 100% goat (caproic acid, coded with 0) to 100% lemon (citral, coded with 100), participants were instructed to rate the baseline stimulus (odorless PG) with the middle marker (coded with 50) on the scale. mix 1: fully masked stimulus, mix 2: incompletely masked stimulus. All comparisons for the quality rating yield a statistically significant difference, however, in this figure only the comparison citral vs mix 1 is depicted (^∗^*p* < 0.05). Error bars display standard deviations. The stimulus “caproic acid” was not presented in the fMRI task.

Regarding the 3 × 5 ANOVA, Mauchly’s test of sphericity was significant and Greenhouse-Geisser Epsilon >0.75. For this reason, Greenhouse-Geisser correction was applied. The effect of scale was not significant [*F*(1.243, 34.793) = 2.701, *p* = 0.102], but the effect of odor was significant [*F*(2.383, 66.725) = 121.614, *p* < 0.001]). The interaction odor × scale reached significance level as well [*F*(3.618, 101.3) = 61.539, *p* < 0.001]. Bonferroni-corrected *t*-tests revealed that citral and the fully masked odor (mix 1) did not differ regarding their pleasantness (*t* = 0.517, *p* = 1) and intensity (*t* = 0.284, *p* = 1), but differed with regard to their quality ratings (*t* = 4.320, *p* = 0.001, mean citral = 71.49, mean mix 1 = 65.52). Bonferroni-corrected *t*-tests also revealed that citral and caproic acid did not differ regarding intensity (*t* = −0.535, *p* = 1), but differed with regard to quality ratings (*t* = −14.612, *p* < 0.001) and pleasantness (*t* = −7.582, *p* < 0.001). Caproic acid was perceived as less pleasant than citral. Intensity, pleasantness and quality ratings are depicted in Figure 2.

**FIGURE 3:**
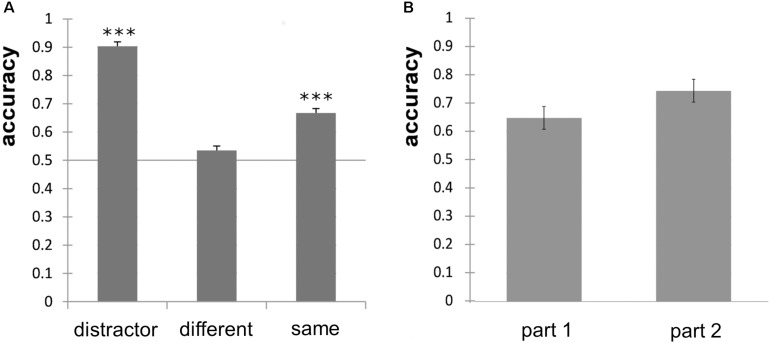
**(A)** Odor discrimination accuracy of the distractor odor pairs, different odor pairs and same odor pairs and significance against chance level of 0.5 (^∗∗∗^*p* < 0.001). **(B)** Non-significant improvement of odor discrimination performance for part 1 (first half) and part 2 (second half) of the experiment; error bars show standard error of the mean.

Overall odor discrimination performance of the subjects differed, ranging from 41.8% correct to 76.3% correct responses. Percentual accuracy values are, however, sensitive to an answering bias. To correct for this bias, we calculated *d*′ and found that only four subjects provided a *d*′ > 1 and were unlikely to guess. We also calculated the response bias *c* and found that 21 participants showed a bias toward the answer “same” in a varying extent and 8 participants showed a small to moderate bias toward the answer “different.” The individual *d*′-values ranged from *d*′ = −0.62 to *d*′ = 1.78 with a mean *d*′ = 0.55. Accuracy for discrimination of “different odor pairs” did not significantly differ from chance level (*p* = 0.11), but accuracy for “same odor pairs” and distractors did (*p* < 0.001, Figure 3A). The number of points achieved did not correlate significantly with performance in the MONEX-40 (*r* = 0.133, *p* = 0.49) or the olfactory discrimination tasks (*r* = −0.15, *p* = 0.50), and the performance in the MONEX-40 and olfactory discrimination tasks did not correlate either (*r* = −0.243, *p* = 0.265). No significant effect of part (part 1, part 2) was revealed in the paired *t*-test (Figure 3B).

### Imaging Data

**FIGURE 4:**
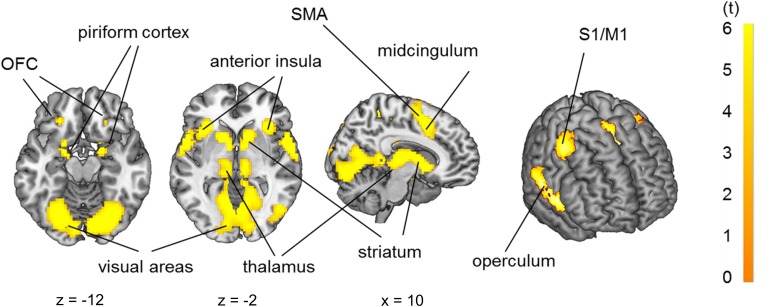
Brain activation due to olfactory stimulation: piriform cortex, orbitofrontal cortex, primary sensorimotor cortices (S1/M1), operculum, insula, striatum, thalamus and visual cortex. *p* < 0.05 FWE-corrected on the brain slices, *p* < 0.001 FWE-corrected on the inflated brain on the right side.

Group analysis (*n* = 29) in a full factorial model revealed bilateral activation of the piriform cortex, orbitofrontal cortex (OFC), posterior-medial frontal cortex (pMFC), anterior insula, operculum (OP), striatum and pallidum, temporal thalamus and primary sensorimotor and visual cortices due to odor stimulation (Figure 4, *p* < 0.05 FWE-corrected, for MNI-coordinates see Supplementary Table 1).

**FIGURE 5:**
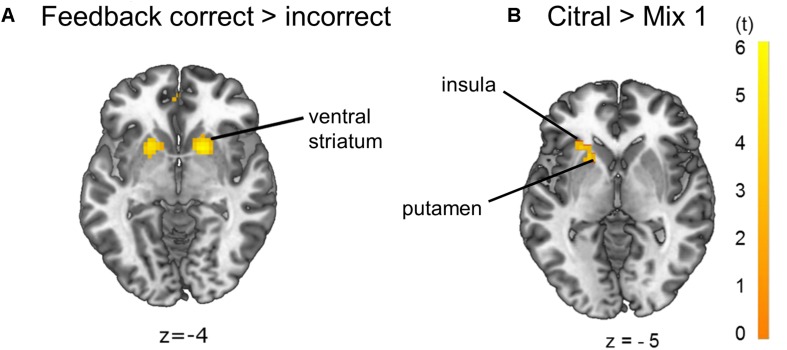
Brain activation due to **(A)** effects of feedback correct > incorrect: ventral striatum and **(B)** effects of citral > mix 1: left putamen and left anterior insula (*p* < 0.05 FWE-corrected on cluster level). The opposite comparison mix 1 > citral did not yield any activation on the *p* < 0.05 FWE-corrected significance level.

Contrasting feedback to odor conditions revealed activation of the anterior cingulate cortex (ACC). Further, contrasting correct to incorrect trials, odor discrimination feedback events revealed a unilateral activation of the angular gyrus, which is part of the inferior parietal lobule (IPL), and a bilateral activation of the ventral putamen, nucleus caudatus and nucleus accumbens, referred to as ventral striatum, which is an area mediating reward value (Delgado, 2007; Diekhof et al., 2012; Jiang et al., 2014; Brand et al., 2016; Wang et al., 2016) (Figure 5A, *p* < 0.05 FWE-corrected, for MNI-coordinates see Supplementary Table 2). The left putamen and anterior insula showed increased activation in the citral condition compared to mix 1 (Figure 5B, *p* < 0.05 FWE-corrected on cluster level, MNI-coordinates *x* = −36, *y* = 14, *z* = 8, cluster of 68 voxels, *t* = 3.65). A bilateral activation of the putamen and ACC, and a unilateral activation of left Broca’s area became evident when the threshold was lowered to *p* < 0.001 uncorrected (MNI-coordinates in Supplementary Table 3). No voxels survived contrasting mix 1 to the citral condition and no voxels survived a linear parametric modulation reflecting a possible increase of activation for the odors at our determined threshold.

**FIGURE 6:**
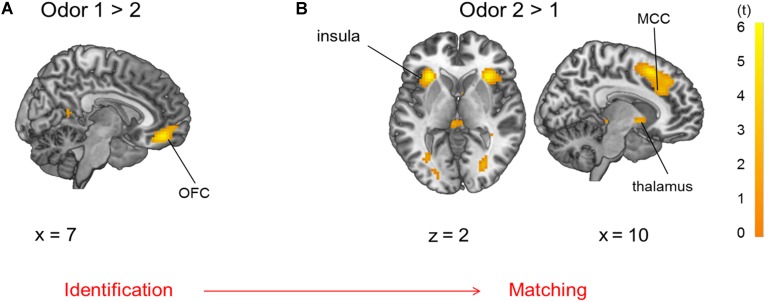
Brain activation due to **(A)** first > second odor presentation: OFC and **(B)** second > first odor presentation: insula, thalamus and mid-cingulate cortex (MCC), both comparisons are *p* < 0.05 FWE-corrected.

During presentation of odor pairs, the effect of the order of odor presentation was also investigated and revealed that the first odor percept was associated with increased activation of the OFC (pars orbitalis and Brodman’s area 32), angular gyrus (IPL), and hippocampus (Figure 6A, *p* < 0.05 FWE-corrected) compared to the second odor. The second odor presentation in contrast to the first odor presentation was accompanied by activation of pMFC, Broca’s area, primary sensorimotor and visual cortices, thalamus and bilateral anterior insula (Figure 6B, *p* < 0.05 FWE-corrected, MNI-coordinates in Supplementary Table 4).

## Discussion

In our daily routine, odor processing is rarely consciously perceived (Stevenson, 2009). However, a subliminal unconscious processing of low odor concentrations might become conscious only when concentrations increase or after some period following exposure (Sela and Sobel, 2010; Mahmut and Stevenson, 2015). In the present paradigm, participants performed above chance level in identifying same odor pairs but only at chance level for the very alike but differing odor stimuli citral and mix 1. By calculating *d*′ we found that 21 of 29 participants showed a bias to choose the answer “same odor pair,” which explains the performance above chance level for the answer “same odor pairs.” During prior hedonic testing, the same group of subjects rated the different odor pairs as isointense and isopleasant, but of differing odor quality. The difference in mean quality ratings does, apparently, not imply that the stimuli were all distinguishable in the discrimination task. The tasks were very different, and during the forced-choice task, where participants had to decide if odors differed or not, many participants could not distinguish citral and mix 1, despite the discriminative results in the quality rating task. Our results are limited to the pleasant and unpleasant odors citral and caproic acid and further research is needed to replicate our results and to investigate other odor pairs. We also did not test for odor identification acuity. Notably, individuals differ in their olfactory acuity and discrimination abilities. Interestingly, task performance did not correlate with olfactory discrimination in the Sniffin’ Sticks task or identification performance in the MONEX-40 task. This indicates that the tests measured distinct yet different components of olfaction and that different skills were required during the tasks.

The imaging data revealed a close link between olfaction and the rewarding nature of the task, as evident by cerebral activation patterns during odor stimulation, which are typically involved in odor processing (Savic, 2002; Royet et al., 2013; Seubert et al., 2013). The olfactory cortex formed a strong cluster with the ventral striatum and anterior insular cortex, suggesting that a rewarding quality is associated with the task during sensory integration of the odor.

The activation of primary sensorimotor cortices during odor stimulation appears to occur as subjects had to provide feedback as a two-choice button press right after the second odor and received feedback on the screen informing them if they were right or wrong immediately after the button press. This may also explain activation in visual areas. Further, the visual activation caused by the scale might be higher than the activation caused by the fixation cross. Apart from that, it is possible that primary sensorimotor activations are observed due to a different weighting, as there were more events with odor than without. Concerning the effect of odor, activation of the thalamus may seem surprising, as the sense of smell is the only sensory system without a specialized thalamic relay (Murakami et al., 2005). Indeed, in contrast to other senses, there is no thalamic nucleus for olfaction which receives input from the spinothalamic tract that projects to the respective primary somatosensory cortex (Kay and Sherman, 2007). Despite this, the olfactory sensation still could trigger this activation, as the olfactory bulb projects to the piriform cortex, which projects to the dorsomedial nucleus of the thalamus (Ongur and Price, 2000) and has reciprocal connections to the OFC that mediate olfactory attention and conscious perception of the odor (Shepherd, 2005; Plailly et al., 2008; Courtiol and Wilson, 2015).

During feedback events activation of the ACC is present, which may reflect the subsequent odor-matching decision (Demanuele et al., 2015). Regarding the effect of correct feedback in contrast to incorrect feedback, increased activation of the ventral striatum was evident, probably reflecting a rewarding experience (Kirsch et al., 2003; Diekhof et al., 2012; Jiang et al., 2014) and learning processes (Seger and Cincotta, 2005). The activation of the ventral striatum provides an overlap with activation evident from the pure odor contrast, correct feedback and citral > mix 1, suggesting a rewarding experience during odor application itself, rather than just during positive feedback trials. Anatomically, the piriform cortex and nucleus accumbens of the ventral striatum are very closely located and also functionally connected (Jiang et al., 2015), and the piriform cortex merges not only information of olfactory perception, but also from higher-order association areas such as the insular cortex (Margot, 2009). Brodmann’s area 39, or more precisely, area PGa of the IPL in the angular gyrus (Caspers et al., 2006) also showed activation in correct feedback trials in contrast versus incorrect feedback trials. This area is involved in inferential reasoning (Goel et al., 1995) and executive control of behavior (Kubler et al., 2006).

Although the different odor pairs were indistinguishable in the forced-choice task, brain activation due to citral differed from that of mix 1. The lemon odor of citral might potentially be more pleasant, maybe due to its unconsciously or consciously recognized odor quality. Another explanation would be that citral fits the concept of a lemon better and might be perceived as being more edible than mix 1 (Jiang et al., 2015). The anterior insula and putamen showed increased activation with citral in contrast to mix 1, despite the fact that an odor mixture is suggested to produce increased activation compared to a single odor (Boyle et al., 2009). The activation possibly depends on the respective intensity of all added components. In fact, odor mixtures may be perceived as a “whole” just as pure odors are (Arzi and Sobel, 2011), depending on configural or elemental processing of an odor mixture (Sinding et al., 2013). Further, the insular cortex is involved in multisensory processing (Legrain et al., 2011) and activation of the putamen is associated with reward (Mizuno et al., 2016). These areas on the right side, not on the left as in our case, are also associated with disgusting smells (Heining et al., 2003), possibly because aversive and pleasant odors are both salient stimuli (Kurth et al., 2010; Legrain et al., 2011; Rolls, 2016).

Evaluating the effect of order, stimuli presented first elicited activation in the OFC, precisely in subgenual Brodman’s area 32 (area s32) and Area PGa (IPL) in contrast to odors presented second in odor pairs, indicating a cognitive and emotional assessment (Palomero-Gallagher et al., 2015) and anticipated sequence processing (Crozier et al., 1999) of the odor stimuli presented first. Area s32 in the rectal gyrus is also involved in taste evaluation and co-activated with areas of executive control (Palomero-Gallagher et al., 2015). Additionally, hippocampal activation was observed, probably due to encoding of the odor stimuli. Odors presented second elicited a more pronounced activation of the anterior insula compared to the first odors, which is consistent with sensory integration and salience processing (Legrain et al., 2011). The pMFC, which is involved in cognitive dissonance (Izuma et al., 2015), Broca’s area, which is connected to smelling familiar odors (Savic and Berglund, 2004) and the thalamus, which is probably involved in olfactory attention and sensory integration of the odor (Plailly et al., 2008), also increased in activation during the second odor stimulus. Possibly, the participants tried to identify the first odor of the odor pair in preparation for the comparison. The second odor of the odor pair was then integrated in a sensory manner and matched to the first one to generate a response. This order effect of stimulus presentation did not depend on odor quality but just on application order of the stimuli. Neuronal activation due to the order effect was stronger than effects of odor quality.

The behavioral data with differing responses concerning odor quality of the pure and the masked odor stimuli is consistent with our fMRI data that reveal differing neuronal processing for the pure and the masked odor. It is our conclusion that differentiation of the pure and masked odor stimuli was possible by using ratings on a dimensional scale rather than a forced-choice task. Possibly the forced choice task is not sensitive enough for measuring the slight difference in odor quality, whereas other tasks are. Our hypothesis that perceptual discrimination can be enhanced in our task using a reinforcing feedback paradigm is not supported by our data. The implications of our findings are, that aversive odors are likely to be satisfactorily addressed by a sufficient amount of a pleasant, masking odor, but that perceptual differences may nonetheless exist, notably on a neuronal level.

## Data Availability Statement

The datasets generated for this study are available on request to the corresponding author.

## Ethics Statement

The studies involving human participants were reviewed and approved by the ethical review board of the Medical Faculty of RWTH Aachen University. The participants provided their written informed consent to participate in this study.

## Author Contributions

RR-R designed the study, acquired and analyzed the data, wrote the manuscript, had full access to all data in the study, and takes the responsibility for the integrity and accuracy of data analysis. HL acquired the data and revised the manuscript. RS analyzed the data and revised the manuscript. JB acquired and analyzed the data, and revised the manuscript. MS and AB revised the manuscript. JF was the guarantor of this work, supervised the study, interpreted results, and revised the manuscript.

## Conflict of Interest

MS was employed by Symrise AG Scent & Care Division, Holzminden, Germany. HL, JB, AB and JF were employed at Sensory Analytics, Fraunhofer Institute for Process Engineering and Packaging IVV, Freising, Germany. The remaining authors declare that the research was conducted in the absence of any commercial or financial relationships that could be construed as a potential conflict of interest.

## Footnotes

1 http://www.fil.ion.ucl.ac.uk/spm
