# Supplementary material for: A Masked Aversive Odor Cannot Be Discriminated From the Masking Odor but Can Be Identified Through Odor Quality Ratings and Neural Activation Patterns
Source: Front Neurosci. 2019 Nov 14;13:1219. doi: 10.3389/fnins.2019.01219 (PMC6868123; doi:10.3389/fnins.2019.01219)
Supplement: Supplementary file 1 [file Table_1.pdf]

Supplementary Material

1 Supplementary Data

PTR-MS validation of the odorant stimuli

The stimulus output of the olfactometer was directly connected to the sample transfer line of the PTR-MS instrument (model hs-PTR-MS, IONICON Analytik GmbH, Innsbruck, Austria), which measured the concentrations of the target odorants continuously throughout a stimulus sequence. Citral was detected at *m/z* 95 and caproic acid at *m/z* 117, as determined in independent analyses of the respective pure compounds. The PTR-MS instrument was operated with a reaction chamber (drift tube) voltage, pressure and temperature of 600 V, 2.2 mbar and 60 °C, respectively, which established an electric field (E) to buffer gas number density (N) ratio of E/N = 132 Td (1 Td = 10^{-21} V m^2). The measurements were performed in multiple ion detection (MID) mode comprising *m/z* 21 (the ^{18}O isotope of the hydronium primary donor ion), measured with a dwell time of 50 ms, and the two target product ions of citral (*m/z* 95) and caproic acid (*m/z* 117) each with a dwell time of 100 ms, resulting in a cycle duration of approximately 250 ms, i.e., a measurement frequency of 4 Hz. Conversion of signal intensity to volume mixing ratio (equivalent to odor concentration) was made using first-order reaction kinetics using a reaction constant of $k = 2\times10^{-9}$ cm^3 s^{-1} (Beauchamp, et al., 2013). The concentrations of the odor stimuli from the olfactometer were observed to gradually increase from start to finish, especially in the stimuli containing higher citral concentrations (citral and mix 1) (cf. Supplementary Material Figure 1). Relative standard deviations of stimulus concentrations for the three mixtures over the 36-min measurement sequence were 9 %, 10 % and 16 % for citral, mix 1 and mix 2, respectively, reflecting a relatively low level of variation. This effect is considered to be within acceptable limits, with such variations not expected to be detectable by the human odor perception.

2 Supplementary Figures and Tables

2.1 Supplementary Tables

Table 1: Effect of odor: Montreal Neurological Institute (MNI)-coordinates, number of voxels exceeding the statistical threshold ($p < 0.05$ FWE-corrected, minimum cluster size of 5 voxels), t-value and anatomical region are reported.

**MNI coordinates**					
**x**	**y**	**z**	**voxels**	**t**	**Brain region**
6	11	50	4745	9.88	R Posterior-Medial Frontal Cortex
3	2	68		8.49	R Posterior-Medial Frontal Cortex
-12	8	-1		8.39	L Pallidum
6	-16	11		4.63	R Thalamus temporal
21	4	-13		4.63	L Piriform Cortex
-57	8	2	2031	9.31	L Rolandic Operculum (Area 44)
-30	23	5		7.98	L Insula Lobe
-60	-13	11		7.93	L Superior Temporal Gyrus (Area OP4 [PV])
63	-4	8	1362	9.13	R Rolandic Operculum (Area OP4 [PV])
60	5	5		9.09	R Rolandic Operculum (Area 44)
45	-4	56		7.94	R Middle Frontal Gyrus
33	26	5	111	8.02	R Insula Lobe
48	-67	2	311	6.40	R Middle Temporal Gyrus (Area hOc5 [V5/MT])
36	-82	8		6.28	R Middle Occipital Gyrus (Area hOc4lp)
24	-82	26		5.78	R Superior Occipital Gyrus (Area hOc3d [V3d])
-24	32	-13	19	6.39	L Inferior Frontal Gyrus (pars Orbitalis [Area Fo3])
-42	-73	11	12	5.19	L Middle Occipital Gyrus (Area hOc5 [V5/MT])
-48	-37	47	11	5.08	L Inferior Parietal Lobule (Area 2 [S1])

Table 2: A) Effect of feedback, B) Effects of feedback correct > incorrect: MNI-coordinates, number of voxels exceeding the statistical threshold (FWE-corrected $p<.05$, minimum cluster size of 5 voxels), t-value and anatomical region are reported.

**MNI coordinates**					
**x**	**y**	**z**	**voxels**	**t**	**Brain region**
A) Effect of feedback					
-3	29	-4	13	5.66	Anterior Cingulate Cortex (Area 33)
B) Effect of feedback correct > incorrect					
18	11	-7	67	6.88	R Putamen
12	17	-4		4.63	R Caudate Nucleus
-21	14	-4	53	6.17	L Putamen
-12	14	4		4.63	L Caudate Nucleus
-45	-67	47	7	4.92	L Angular Gyrus (Area PGa [IPL])

Table 3: Effects of odor citral > mix 1: MNI-coordinates, number of voxels exceeding the statistical threshold ($p < 0.001$ uncorrected, minimum cluster size of 5 voxels), t-value and anatomical region are reported.

**MNI coordinates**					
**x**	**y**	**z**	**voxels**	**t**	**Brain region**
21	11	-7	22	4.99	R Putamen
-36	14	8	68	3.65	L Insula Lobe
-3	20	23	9	4.33	L Anterior Cingulate Cortex (Area 33)
12	38	26	10	4.09	R Anterior Cingulate Cortex
-60	5	23	8	3.80	L Precentral Gyrus (Area 44)
-60	5	14	8	3.64	L Postcentral Gyrus (Area 44)

Table 4: A) Effects of sequence odor 1 > odor 2. B) Effects of sequence odor 2 > odor 1: MNI-coordinates, number of voxels exceeding the statistical threshold (FWE-corrected $p < 0.05$, minimum cluster size of 5 voxels), t-value and anatomical region are reported.

MNI coordinates					
**x**	**y**	**z**	**voxels**	**t**	**Brain region**
A) Effect of sequence odor 1 > odor 2					
-3	41	-16	200	9.97	L Rectal Gyrus (Area s32)
-45	-67	38	103	8.38	L Angular Gyrus (Area PGa [IPL])
-33	23	53	62	7.83	L Middle Frontal Gyrus
-63	-10	-10	55	7.58	L Middle Temporal Gyrus (Area TE 3)
-63	-49	-1	26	7.17	L Middle Temporal Gyrus
24	-16	-13	8	7.12	R Hippocampus
60	-4	-22	13	6.38	R Middle Temporal Gyrus
-42	32	-16	8	6.35	L Inferior Frontal Gyrus (pars Orbitalis)
-3	-58	26	49	6.12	L Precuneus
6	-52	14	4	5.98	R Precuneus
48	-67	32	5	5.73	R Angular Gyrus (Area PGp [IPL])
B) Effect of sequence odor 2 > odor 1					
6	17	53	626	10.66	R Posterior-Medial Frontal Cortex
-9	-4	56	626	9.01	L Posterior-Medial Frontal Cortex
-9	17	44	626	8.82	L Superior Medial Gyrus
36	26	8	224	10.53	R Inferior Frontal Gyrus (pars Triangularis)
-30	26	2	223	10.27	L Insula Lobe
-42	-34	47	556	9.03	L Postcentral Gyrus (Area 2 [S1])
-33	-31	50	556	9.02	L Postcentral Gyrus (Area 4p)
-27	-52	47	556	8.56	L Inferior Parietal Lobule (Area hIP3 [IPS])
-24	-76	-4	286	8.82	L Fusiform Gyrus (Area hOc4v [V4(v)])
24	-76	-4	393	8.00	R Lingual Gyrus (Area hOc4v [V4(v)])
27	-73	8	393	7.48	R Calcarine Gyrus (Area hOc1 [V1])
-42	-1	35	46	8.35	L Precentral Gyrus
57	20	32	156	7.60	R Inferior Frontal Gyrus (pars Opercularis [Area 45])
39	17	29	156	7.47	R Inferior Frontal Gyrus (pars Triangularis)
51	8	32	156	6.62	R Precentral Gyrus (Area 44)
6	-25	-4	24	6.24	R Thalamus

2.2 Supplementary Figures

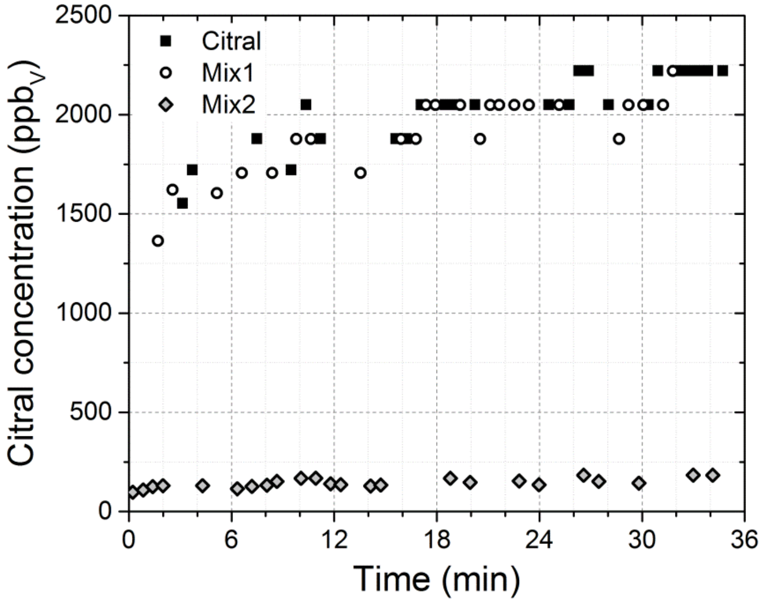

Figure 1: Concentrations of citral from the three stimuli (citral, mix 1 and mix 2) produced by the olfactometer over the 36-min presentation sequence.

References

Beauchamp, J., J. Herbig, J. Dunkl, W. Singer, and A. Hansel. 2013. 'On the performance of proton-transfer-reaction mass spectrometry for breath-relevant gas matrices', *Measurement Science and Technology*, 24: 125003.
